# Comparing diagnostic tests and biomarkers: trials in people with discordant test results

**DOI:** 10.1186/1745-6215-12-S1-A19

**Published:** 2011-12-13

**Authors:** Richard Hooper, Karla Díaz-Ordaz, Andrea Takeda, Khalid Khan

**Affiliations:** 1 Centre for Primary Care & Public Health, Blizard Institute, Queen Mary University of London, London E1 2AB, UK

## Objectives

Diagnostic tests are traditionally compared for accuracy against a gold standard, but there is growing interest in tests (or biomarkers) used to guide treatment choices rather than specifically to diagnose. The question of whether health outcomes are better using one test or another in the same population can be answered with a randomised trial. It has been suggested that an efficient approach to trialling is to give both tests to all participants, and randomise and follow up those with discordant results. We describe how to plan and analyse such a trial, and consider its efficiency compared with a conventional trial design.

## Methods

We investigated two estimates of risk difference for a binary outcome: one based on analysing outcomes as if from a conventional trial (the trial estimate), and one which combined estimates of different parameters in the manner of a decision analysis (the decision analysis estimate). From theory we derived the bias and standard error of each. We also considered the impact of randomising participants to a testing strategy before the tests are administered rather than after.

## Results

The trial estimate and decision analysis estimate are both unbiased. Using the decision analysis estimate (but not the trial estimate) the same precision is achieved by randomising before testing as by randomising after. Giving both tests to all participants means fewer need to be recruited: in one example from the literature the proposed design was nearly four times more efficient in this sense than a conventional trial design.

## Conclusions

We suggest the term “randomised discordance trial” for the design we have described. A discordance trial offers an efficient way to compare two diagnostic tests or biomarkers. Randomising before testing avoids selection bias. We have derived formulae for calculating sample size and for estimating risk difference and standard error using this design.

